# Pharmacokinetic, Ambulatory, and Hyperthermic Effects of 3,4-Methylenedioxy-*N*-Methylcathinone (Methylone) in Rats

**DOI:** 10.3389/fpsyt.2017.00232

**Published:** 2017-11-17

**Authors:** Kristýna Štefková, Monika Židková, Rachel R. Horsley, Nikola Pinterová, Klára Šíchová, Libor Uttl, Marie Balíková, Hynek Danda, Martin Kuchař, Tomáš Páleníček

**Affiliations:** ^1^Department of Experimental Neurobiology, National Institute of Mental Health, Klecany, Czechia; ^2^First Faculty of Medicine, Institute of Forensic Medicine and Toxicology, Charles University and General University Hospital in Prague, Prague, Czechia; ^3^Third Faculty of Medicine, Charles University in Prague, Prague, Czechia; ^4^Department of Physiology, Faculty of Science, Charles University, Prague, Czechia; ^5^Forensic Laboratory of Biologically Active Compounds, Department of Chemistry of Natural Compounds, University of Chemistry and Technology Prague, Prague, Czechia

**Keywords:** methylone, bk-3,4-metyhlenedioxymethamphetamine, nor-methylone, novel psychoactive substances, cathinones, behavior, pharmacokinetics, metabolites

## Abstract

Methylone (3,4-methylenedioxy-*N*-methylcathinone) is a synthetic cathinone analog of the recreational drug ecstasy. Although it is marketed to recreational users as relatively safe, fatalities due to hyperthermia, serotonin syndrome, and multi-organ system failure have been reported. Since psychopharmacological data remain scarce, we have focused our research on pharmacokinetics, and on a detailed evaluation of temporal effects of methylone and its metabolite nor-methylone on behavior and body temperature in rats. Methylone [5, 10, 20, and 40 mg/kg subcutaneously (s.c.)] and nor-methylone (10 mg/kg s.c.) were used in adolescent male Wistar rats across three behavioral/physiological procedures and in two temporal windows from administration (15 and 60 min) in order to test: locomotor effects in the open field, sensorimotor gating in the test of prepulse inhibition (PPI), and effects on rectal temperature in individually and group-housed rats. Serum and brain pharmacokinetics after 10 mg/kg s.c. over 8 h were analyzed using liquid chromatography mass spectrometry. Serum and brain levels of methylone and nor-methylone peaked at 30 min after administration, both drugs readily penetrated the brain with serum: brain ratio 1:7.97. Methylone dose-dependently increased overall locomotion. It also decrease the amount of time spent in the center of open field arena in dose 20 mg/kg and additionally this dose induced stereotyped circling around the arena walls. The maximum of effects corresponded to the peak of its brain concentrations. Nor-methylone had approximately the same behavioral potency. Methylone also has weak potency to disturb PPI. Behavioral testing was not performed with 40 mg/kg, because it was surprisingly lethal to some animals. Methylone 10 and 20 mg/kg s.c. induced hyperthermic reaction which was more pronounced in group-housed condition relative to individually housed rats. To conclude, methylone increased exploration and/or decreased anxiety in the open field arena and with nor-methylone had short duration of action with effects typical for mixed indirect dopamine–serotonin agonists such as 3,4-metyhlenedioxymethamphetamine (MDMA) or amphetamine. Given the fact that the toxicity was even higher than the known for MDMA and that it can cause hyperthermia it possess a threat to users with the risk for serotonin syndrome especially when used in crowded conditions.

## Introduction

Methylone (3,4-methylenedioxy-*N*-methylcathinone, also known as MDMC, bk-MDMA, M1) belongs to the group of new psychoactive substances called synthetic cathinones often also termed as β-keto amphetamines or the new generation of designer phenethylamines (1). This β-keto analog of 3,4-methylenedioxy-methamphetamine (MDMA; “ecstasy”) was first synthetized in 1996 as an antidepressant and anti-Parkinsonian agent (2) but was never used for therapeutic purposes; instead, it gained popularity as a recreational “legal high” owing to its MDMA-cocaine-like effects (3). Methylone users describe their subjective experience as feeling stimulated, with a great need to socialize, spiritual, and empathic connection. Methylone first appeared in 2004 on the illicit drug market (in the Netherlands) and quickly became commonly available and easily obtainable (4), leading to extensive abuse worldwide (5). European Monitoring Center for Drugs and Drug Addiction and Europol have monitored methylone since 2005 (6) and starting in 2011, methylone was reclassified as Schedule I under the Controlled Substance Act in the Unites States (7). In the United Kingdom, methylone has been illegal since 2010. There have been a number of reports of methylone toxicity and even fatal overdoses have been registered. The causes of death include hyperthermia where body temperature elevated up to 41.7°C as a core symptom of serotonin syndrome, metabolic acidosis, and multi-organ system failure (8, 9). Even though the popularity of methylone among users as well as its availability on the gray/black market is widespread, the relevant scientific data are still relatively scarce, and there are no published data on behavioral effects of nor-methylone. Therefore, we investigated effects of these substances on behavior, pharmacokinetics, and body temperature.

Methylone only differs from MDMA by the presence of a ketone at the benzylic position. Based on their structural similarity, and, in turn, similar mechanism of action, comparable effects on behavior, and neurochemistry could be postulated (10). *In vitro* neuropharmacological studies in rat’s brain, synaptosomes reported methylone as a non-selective inhibitor of the dopamine, norepinephrine, and serotonin transporters (DAT, NET, and SERT, respectively). Further, methylone *via* blocking re-uptake evokes high-releasing activity of all monoamines (dopamine, norepinephrine, and serotonin) (10, 11). The ratio of DAT:SERT inhibition is 3.3 which suggests that methylone has a high-abuse potential similar to cocaine (DAT:SERT ratio 3.1) (3, 12) as has been also confirmed in behavioral tests (13, 14). On the other hand, in discrimination studies, methylone substituted for MDMA indicating a similar profile (and subjective effects) to this serotonergic compound (15). Thus far, behavioral research has shown that methylone increases locomotor activity, an effect which was inhibited by both dopamine (D2) or serotonin (5-HT_2A_) receptor antagonists (16–19). Repeated administration of methylone, similar to acute effects of other MDMA-like compounds, induced hyperthermia in rats: an effect typically mediated by serotonin and typically associated with acute toxicity of sertonergic drugs (20–22). On the other hand, simultaneous study of Javadi-Paydar et al. (19) showed no change in body temperature in rats treated by methylone. Methylone is the subject of extensive metabolism in the liver at cytochrome P450 (isoenzymes CYP2D6, CYP1A2, CYP2B6, and CYP2C19) with major primary metabolite nor-methylone, which are subsequently excreted to urine unchanged or in their conjugated forms (23, 24). Other metabolites (dihydroxymethcathinone, *N*-hydroxy-methylone, and dihydro-methylone) were also detected and to date no information about the biological activity of these have been published (23).

Our primary intention was to describe in detail temporal profile of methylone’s effects in behavioral tests alongside pharmacokinetics and the effects on body temperature to evaluate its eventual serotonergic toxicity related to hyperthermia. An added value of our study was in the evaluation of the effects of nor-methylone as the primary metabolite in the same series of behavioral tasks. Finally, since we have performed series of experiments in our laboratory with related cathinones this allows us to make indirect comparisons between those (21, 25–27).

In the behavioral study, to test its effects on locomotion, exploratory activity, anxiety, and stereoytypy the open field test was used, further on effects on sensorimotor gating indicative of its psychomimetic potential were tested in the test of prepulse inhibition of acoustic startle response (PPI ASR) (28). To cover the peak effects as well as possible late onset of changes in these tests, we tested both paradigms in two temporal windows (15 and 60 min) after drug administration. To link the behavioral data to serum and brain levels of methylone, the samples for pharmacokinetics were collected from animals involved in behavioral experiments. According to its structural and pharmacological similarity with MDMA and cocaine, we hypothesized that it will have similar behavioral profile (stimulatory and disruptive) but shorter duration of action. Finally, as it has been shown previously for other compounds, environmental factors, especially an effect of “individually/group-housed” rats (e.g., people dancing in a crowded clubs) significantly increase the risk of hyperthermic reaction, we also tested the effect of isolation and aggregation on hyperthermic effects of methylone.

## Materials and Methods

### Animals

All animals were male Wistar rats (Hannover breed, obtained from Konárovice, Czech Republic) weighing 200–250 g and aged 8 weeks at the start of testing. Rats were housed two per cage under controlled temperature (22 ± 2°C) and humidity (30–70%) with food pellets and water freely available. Lights were on from 6:00 to 18:00 h and all experiments were carried out between 7:00 and 13:00 h, except the temperature study where the test lasted until 17:00 h. Animals were allowed 7–10 days to habituate to laboratory conditions before being used in experiments, during which they were weighed twice and handled four times. All behavioral experiments were conducted in the same standard conditions (temperature and humidity) as in the animal housing facility. Each experimental group for behavioral and temperature studies included 10 animals and each animal was tested only once with the exception that (to reduce animal use) rats from behavioral experiments were used for subsequent pharmacokinetic sampling [for pharmacokinetic studies, *n* = 8 (for methylone) and *n* = 5 (for nor-methylone) per experimental group]. All procedures were conducted in accordance with the principles of laboratory animal care of the National Committee for the Care and Use of Laboratory Animals (Czech Republic), and according to Guidelines of the European Union (86/609/EU). The protocol was approved by the National Committee for the Care and Use of Laboratory Animals (Czech Republic) under the number: MEYSCR-27527/2012-31.

### Drugs and Chemicals

3,4-Methylenedioxy-*N*-methylcathinone (methylone) was purchased *via* the internet and subsequently purified and converted to a hydrochloride (HCl) by Alfarma s.r.o. (Czech Republic). The resulting methylone was certified to be of 99.18% purity (analyzed by infrared spectroscopy) and also served as a reference standard for pharmacokinetic analyses using liquid chromatography. Nor-methylone was synthesized in the Forensic Laboratory of Biologically Active Substances (University of Chemistry and Technology Prague, Czech Republic) in a purity of 99.18%. Internal standards for quantitative liquid chromatography/mass spectrometry (LC/MS) assays were deuterated MDA-D2. HCl with the purity 99.7% (Lipomed, Inc., Switzerland). Reference standards for confirmation of metabolites by LC/HRMS (high-resolution mass spectrometry) and gas chromatography/mass spectrometry were synthesized with purity within 97.5–89.3% (Institute of Chemical Technology, Department of Organic Chemistry, Prague). β-Glucuronidase type HP-2 from Helix Pomatia, EC 3.2.1.31 (184,973 U/ml) was purchased from Sigma-Aldrich, Prague. Extraction columns Bond Elut Certify 50 mg/3 ml were supplied by Labio s.r.o., Olomouc. Other chemicals used for laboratory purposes were of analytical grade purity. Methylone was stored in dry and dark place and dissolved in physiological saline (0.9% NaCl) immediately before experiments.

### Dosage

The methylone doses used in the present study were estimated according to the reported usage by humans and according to our previous studies with entactogens MDMA, para-methoxymethamphetamine (PMMA), and 2C-B (4-bromo-2,5-dimethoxyphenylethylamine). Doses were selected to range from those that: (1) at the lower end are close to those used by humans, to (2) higher doses that might produce significant acute non-lethal toxicity, and (3) with respect to our previous analogous experiments with MDMA, PMMA, and 2C-B (21, 29–31). The treatment range for methylone was set to be 5, 10, 20, and 40 mg/kg, nor-methylone at 10 mg/kg for behavioral experiments. In behavioral experiments (open field, PPI ASR) nor-methylone was tested only in 15 min testing onset. Doses for pharmacokinetic [10 mg/kg subcutaneously (s.c.)] and temperature experiments (10 and 20 mg/kg s.c.) were selected according to the inherent sensitivity of the analytical LC/HRMS procedure utilized and according to the effectiveness in behavioral tasks (effects body temperature). For the pharmacokinetic study, a single bolus of methylone 10 mg/kg s.c. was administered, subsequently animals were decapitated after 30, 60, 120, 240, or 480 min. Additional pharmacokinetic data with the same design were also obtained for nor-methylone 10 mg/kg s.c. Separated sera and whole brains were kept at −20°C until the toxicological analyses. Both drugs were dissolved in a volume of 2 ml/kg and administered s.c. (for comparability with previous studies) in all cases.

### Pharmacokinetic Analyses

#### Determination of Methylone and Nor-Methylone Levels in Serum and Brain Sample Using LC/HRMS

##### Serum Pre-Treatment

0.2 ml of rat serum was fortified with the internal standard mephedrone-D7 and nor-mephedrone-D7 in methanolic solution (in an amount with respect to the level of methylone and nor-methylone in assayed samples) and 0.5 ml of a 0.1 M phosphate buffer (pH 6) in a labeled tube.

##### Brain Pre-Treatment

250 mg of brain was homogenized with 5 ml methanol and the internal standard methylone-D3 (in an amount with respect to the methylone levels in samples). The specimen was then ultrasonicated for 20 min and after supernatant separation by centrifugation, the supernatant was transferred into a clean labeled tube and evaporated to dryness. The residue was reconstituted in a 0.1 M phosphate buffer (pH 6). Solid phase extraction of methylone in pre-treated samples: a pre-treated sample of serum or brain with the buffer and internal standard was loaded onto a Bond Elut Certify cartridge previously conditioned with 0.5 ml of a 0.1 M phosphate buffer (pH 6). After application of a pre-treated sample, the cartridge was washed with 0.5 ml of distilled water, 0.5 ml of 0.1 M HCl, and 0.5 ml of CH_3_OH/H_2_O (1/1, v/v) and then dried by air for 5 min. The analyses were eluted three times with 0.5 ml of a freshly prepared mixture of dichloromethane/2-propanol/ammonium hydroxide (25%), 80/20/4, v/v/v. The eluate was gently evaporated to dryness under a stream of air at 40°C and then dissolved into mobile phase for LC/HRMS analysis.

#### Determination of 4-Hydroxy-3-Methoxymethcathinone Metabolite in Serum

4-hydroxy-3-methoxymethcathinone (4-OH-3-MeO-MC) was identified in rat serum samples according its exact mass. The calculated [M + H^+^] *m*/*z* for 4-OH-3-MeO-MC (C_11_H_16_NO_3_) was 210.1125. Any peak at the same *m*/*z* was found in blank rat sera.

#### LC/HRMS Conditions

The analyses were performed using Dionex Ultimate 3000 UHPLC coupled to an Exactive Plus-Orbitrap MS (ThermoFisher Scientific, Bremen, Germany) equipped with an HESI-II source. The chromatographic analyses of serum and tissue samples were performed using a Kinetex PFP 100 A (50 mm × 2.1 mm, 2.6 mm) and Security Guard Cartridge PFP 4 mm × 2.0 mm (Phenomenex) with a flow rate of 400 ml/min, gradient elution with 10 mM ammonium formate in 0.1% of formic acid as the mobile phase B. Gradient 0 min 5%, 4 min 45% B, and 5–6 min hold at 95%. The MS conditions were: full MS in a scan range of 50–500 *m*/*z* with positive electrospray ionization, resolution of 70,000 FWHM (scan speed 3 Hz), spray voltage of 3 kV, and ion transfer capillary temperature of 320°C.

### Behavioral Procedures

#### Open Field

Open field testing was conducted in a temperature 22 ± 2°C, sound-proof, and evenly lit chamber with low levels of light intensity. The open field apparatus comprised a black square plastic open field arena (68 cm × 68 cm) with walls (30 cm high). At the beginning of each test, the rat was placed individually into the center of arena 15 or 60 min after drug administration and allowed to move about the arena freely for 30 min. The apparatus was cleaned with 50% ethanolic solution to avoid odors after each test. Behavioral activity was registered by an automatic video tracking system (EthoVision Color Pro v. 3.1.1, Noldus, the Netherlands).

Dependent variables were (i) total locomotor activity over 30 min, (ii) locomotor activity in 5 min intervals, (iii) time spent in the center of the arena and (*T*_center_), and (iv) thigmotaxis (i.e., likelihood of appearance in the periphery). For evaluation of time spent in the center and thigmotaxis the arena was virtually divided into 5 × 5 grid of identical square zones with 16 being located on the periphery and 9 centrally. Time spent in the center of the arena is the sum of time spent in the nine central zones (*T*_center_ = Σtime_1–9_). Thigmotaxis indicates probability of appearances in peripheral zones (*f*; the total number of appearances of the animal in each zones) and is calculated as Σ*f*_peripheral zones_ divided by Σ*f*_all zones_.

#### PPI of ASR

Prepulse inhibition took place in startle chambers (SR-LAB, San Diego Instruments, CA, USA), each containing sound-proof and evenly lit enclosure, high-frequency loudspeaker (produced background noise at 75 dB and all acoustic stimuli), and Plexiglas stabilimeter (8.7 cm inner diameter). A piezoelectric accelerometer detected amplitudes of the startle responses which were digitized for subsequent analysis. 15 or 60 min prior to test rats were administered with methylone, nor-methylone, or vehicle. The experimental design was according to previous studies (21, 27, 29) and consisted of acclimatization and two sessions.

Acclimatization performed 2 days before test, drug-free rats were habituated in 5 min session with five presentations of pulse alone stimuli (115 dB/20 ms) over background white noise (75 dB). Startle data were not recorded for acclimatization.

The test started with a habituation period lasting 5 min in the startle chamber in which a 75 dB background white noise was continuously presented. The PPI test followed with 72 trials in all with an inter-trial interval (ITI) of 4–20 s (mean ITI: 12.27 s). Six 125 dB/40 ms duration pulse alone trials were then delivered to establish baseline ASR. Following this, 60 trials of the following were presented in a pseudorandom order: (A) pulse alone: 40 ms 125 dB; (B) prepulse-pulse: 20 ms 83 or 91 dB prepulse, a variable (30, 60, or 120 ms) inter-stimulus interval (mean 70 ms), then 40 ms 125 dB pulse; and (C) 60 ms no stimulus. Finally, six pulse alone trials were delivered. Habituation was calculated by the percentage reduction in ASR from the initial six baseline trials, to the final six trials. The PPI was calculated as [100 − (mean response for the prepulse − pulse trial/startle response for the single pulse trials) × 100].

#### Rectal Temperature

Rats were divided into two groups: rats housed individually and five animals per cage. These two conditions compared isolated and group-housed conditions and their interaction of drug on body temperature. Rectal temperature was measured using a digital thermometer; every temperature measurement lasted 10 s and rat was momentarily immobilized in a Plexiglas tube. The first measurements were taken every hour at 7:00 until 9:00 h and were taken under drug-free conditions. Methylone or vehicle was administered at 9:00 h and temperature was recorded every half hour until 11:00 h. Thereafter, temperature was recorded at hourly intervals until 17:00 h.

### Design and Statistical Analysis

All statistical analyses were conducted using IBM SPSS version 22. For the open field, PPI, and temperature analyses, factorial designs for later analysis with analysis of variance (ANOVA) were used.

Significant main effects and interactions ANOVAs were followed with pairwise comparisons using independent *t*-tests. For repeated measures ANOVAs, where Mauchly’s test of sphericity was significant (and Mauchley’s *W* < 0.75), Greenhouse–Geisser corrected statistics are reported. For independent *t*-tests, where Levene’s test for equality of variances was significant, statistics corrected for unequal variances are given *p* < 0.05 (two tailed) was considered the minimal criterion for statistical significance. For multiple comparisons, *t*-tests were used with Bonferroni correction. Nor-methylone was not included in ANOVA analyses (only one time of administration was tested, 15 min) and data were analyzed using additional independent *t*-test.

## Results

### Pharmacokinetics of Methylone and Nor-Methylone in Serum and Brain Tissue

**Figure 1:**
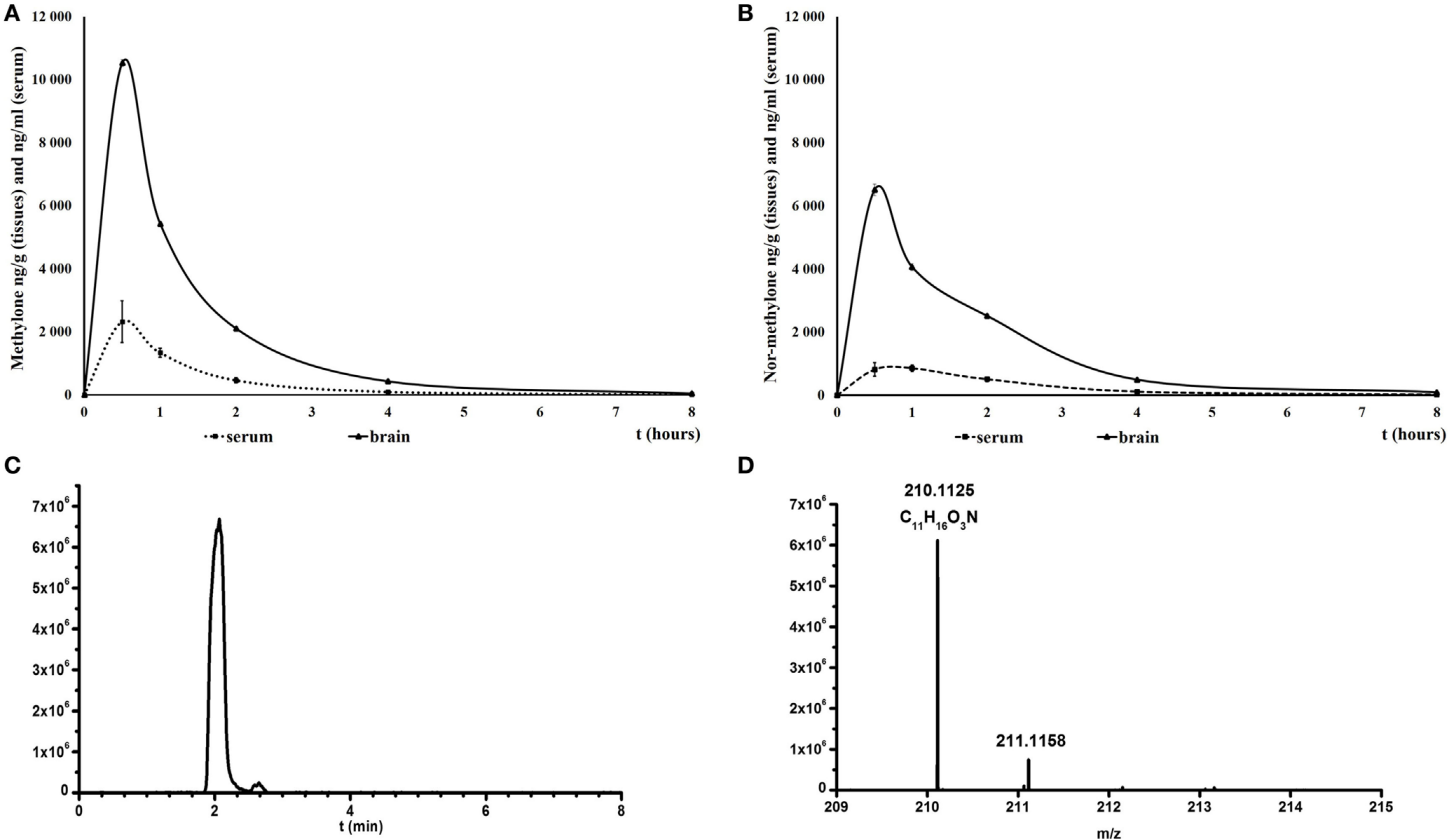
Mean concentrations of methylone **(A)** and nor-methylone **(B)** in serum (nanogram per milliliter) and brain (nanogram per gram) over 8 h after subcutaneously administration of methylone 10 mg/kg and nor-methylone 10 mg/kg, respectively. Symbols represent means and vertical bars SEMs. Second panel represents extracted ion chromatogram of 4-OH-3-MeOH-MC taken at *m*/*z* 210.1125 in rat serum **(C)** and the measured [M + H^+^] *m*/*z* in full spectrum **(D)**.

For methylone, maximum brain and serum concentration were attained within 30 min after the drug administration. The influx into the brain was not delayed and the concentration of methylone in brain was approximately five times higher than serum levels throughout the experiment (serum:brain ratio during the peak was 1:4.54; Figure 1A). Serum levels of nor-methylone were also quantified after methylone administration; they peaked 30 min later than methylone (1 h after administration) and reached about 20% of methylone levels (350 ng/ml). The second most abundant metabolite identified in the serum was 4-OH-MeO-MC (4-hydroxy-3-methoxymethcathinone) (Figures 1C,D), quantification was not possible because of lack of reference standard at the time of analysis. 4-OH-MeO-MC peaked quickly at 30 min after administration of methylone, the peak had bigger area under the curve and then quickly disappeared and nor-methylone became the most abundant at later time points evaluated. After nor-methylone administration, the maximum serum concentrations were reached between 30 min and 1 h, the brain peak appeared at 30 min; the maximum levels were approximately one half when compared with methylone and serum:brain ratio during the peak was 1:7.97 (Figure 1B).

### Acute Toxicity

Rats, treated with methylone 40 mg/kg, were tested only in 15 min testing onset in PPI because after 2 h after administration seven rats no longer produced much behavior only lying on the floor. After 5 h rats began moving around the home cage again, however, mortality occurred within 24 h after injection in six rats. In open field testing, only one rat died within 24 h after administration of methylone (40 mg/kg). Behavioral testing at 60 min after administration was not performed since 40 mg/kg was lethal to some animals.

### Locomotor Activity in the Open Field

Trajectory length was evaluated using 4 × 2 × 6 mixed factorial ANOVAs with drug treatment (methylone at 5, 10, or 20 mg/kg versus vehicle) and time of administration (15 and 60 min) as independent factor, and blocks (5 min interval) as a repeated measures factor. Mauchly’s test of sphericity was significant and Greenhause–Geisser correction are presented for repeated measures, Mauchly’s *W*(14) = 0.21, *p* < 0.001. Degrees of freedom were rounded to whole number for presentational purposes.

**Figure 2:**
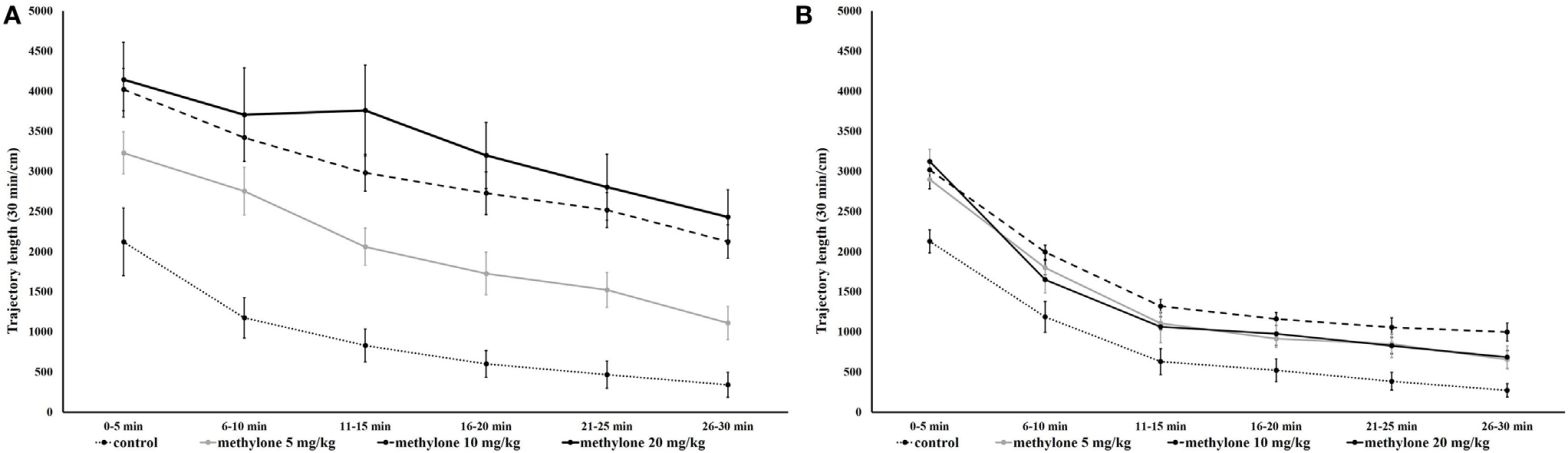
Mean traveled distance within 5-min interval 15 min **(A)** and 60 min **(B)** after administration of methylone (5, 10, and 20 mg/kg). Symbols represent means and vertical bars SEMs.

Analyses produced significant main effects of drug treatment [*F*_(3, 71)_ = 22.43, *p* < 0.001], time of administration [*F*_(1, 71)_ = 50.68, *p* < 0.001], and blocks [*F*_(3, 211)_ = 188.43, *p* < 0.001]. In addition, there was a significant time of administration × drug treatment interaction [*F*_(3, 71)_ = 8.37, *p* < 0.001] and a significant time of administration × blocks interaction [*F*_(3, 211)_ = 6.81, *p* < 0.001] no other interactions were observed (Figure 2).

**Figure 3:**
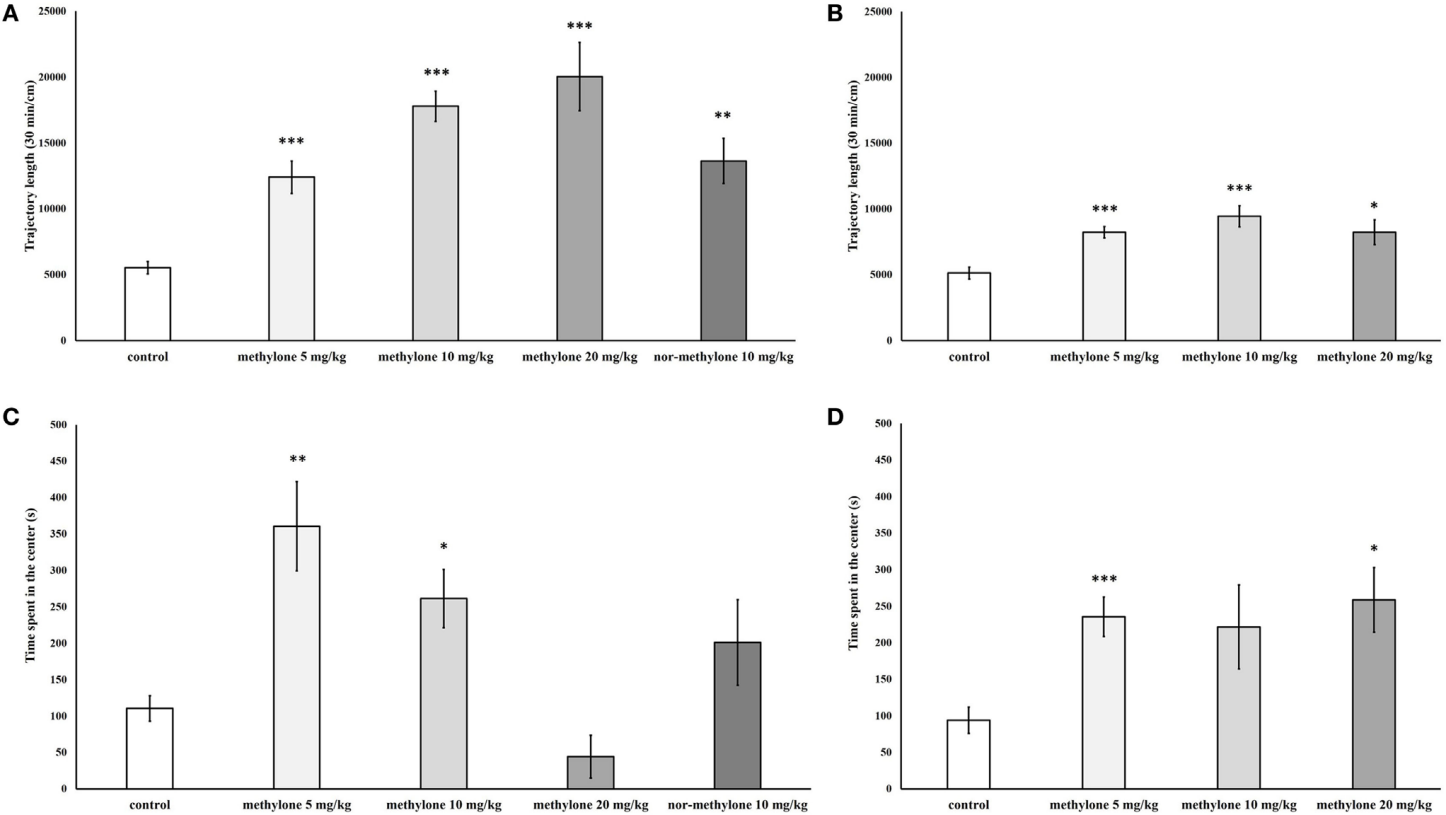
Total locomotion measured 15 min **(A)** and 60 min **(B)** after methylone (5, 10, and 20 mg/kg) and nor-methylone (10 mg/kg) administration. Second panel represents mean *T*_center_ measured 15 min **(C)** and 60 min **(D)** after methylone (5, 10, and 20 mg/kg) and nor-methylone (10 mg/kg) administration. Columns represent means and vertical bars SEMs. ***p < 0.001, **p < 0.01, and *p < 0.05.

Since no interaction between drug treatment × blocks was observed further pairwise comparisons using independent *t*-tests were used to explore the significant on total trajectory length; these revealed that compared with vehicle all three doses of methylone significantly increased locomotion at 15 min [minimum *t*(13) = 5.17, *p* < 0.001; Figure 3A] as well as at 60 min [minimum *t*(12) = 2.99, *p* < 0.05; Figure 3B]. The increase at 60 min was much less pronounced.

Nor-methylone 10 mg/kg compared with vehicle significantly increased total locomotion at 15 min after administration [*t*(18) = 4.57, *p* < 0.01], by contrast, compared nor-methylone to methylone 10 mg/kg there was no significant difference (Figure 3A).

### Thigmotaxis and Time Spent in the Center Part of the Apparatus

Thigmotaxis and *T*_center_ of arena were each analyzed with 4 × 2 ANOVAs with drug treatment and time of administration as independent factors.

#### *T*_center_

There was only a significant main effect of drug treatment [*F*_(3, 71)_ = 9.82, *p* < 0.001] and a significant interaction of time of administration × drug treatment [*F*_(3, 71)_ = 6.37, *p* < 0.001].

Independent *t*-tests showed that methylone at all doses and both times of administration, except 20 mg/kg 15 min and 10 mg/kg at 60 min, significantly increased *T*_center_ [minimum *t*(11) = 3.44, *p* < 0.05]. Nor-methylone had no effect on *T*_center_ (Figures 3C,D).

#### Thigmotaxis

The main effect of time of administration [*F*_(1, 71)_ = 22.15, *p* < 0.001], drug treatment [*F*_(3, 71)_ = 7.38, *p* < 0.001] as well as their interaction [*F*_(3, 71)_ = 14.89, *p* < 0.001] were significant.

Methylone 20 mg/kg at 15 min before measurement significantly increased thigmotaxis [*t*(18) = 7.93, *p* < 0.001]. In contrast, at 60 min 5 and 20 mg/kg decreased it [minimum *t*(18) = 2.68, *p* < 0.05], while nor-methylone had no effect on this parameter (Table 1).

**Table 1:** Mean thigmotaxis measured 15 and 60 min after methylone (5, 10, and 20 mg/kg) and nor-methylone (10 mg/kg) administration.

Drug treatment						
Measure	Admin time	Vehicle	5 mg/kg	10 mg/kg	20 mg/kg	Nor-methylone
Thigmotaxis	15 min	0.82 (0.01)	0.78 (0.02)	0.83 (0.02)	0.97 (0.02)	0.84 (0.04)
	60 min	0.81 (0.01)	0.75 (0.02)	0.82 (0.03)	0.74 (0.02)	xxx

*Numbers represent means and in brackets are shown SEMs*.

### Prepulse Inhibition

Habituation, ASR, and PPI data were each analyzed with 4 × 2 independent ANOVAs with drug treatment (methylone at 5, 10, and 20 mg/kg versus vehicle) and time of administration (15 and 60 min) as independent factors.

Habituation data showed a significant main effect of time of administration on habituation [*F*_(1, 72)_ = 8.17, *p* < 0.01] but no interaction was observed. Independent *t*-tests revealed that methylone compared with vehicle did not affect habituation at any of the doses tested (Table 2).

**Table 2:** The effect of methylone (5, 10, and 20 mg/kg) and nor-methylone (10 mg/kg) on acoustic startle response (ASR) and habituation.

Drug treatment						
Measure	Admin time	Vehicle	5 mg/kg	10 mg/kg	20 mg/kg	Nor-methylone
ASR (*arbitrary units*)	15 min	183.4 (60.1)	79.9 (12)	237.2 (36)	188.5 (23.4)	172 (33)
	60 min	157.5 (36.2)	125 (26.5)	157.2 (34.5)	145 (17.2)	xxx
Percentage habituation	15 min	40.4 (10.9)	25.7 (13.4)	19.4 (9)	35.7 (8)	35.6 (7.1)
	60 min	67.1 (6.1)	50.8 (8.4)	43.3 (8.6)	47.2 (6.9)	xxx

*Numbers represent means and in brackets are shown SEMs*.

Acoustic startle response data showed a significant main effect of drug treatment [*F*_(3, 72)_ = 2.83, *p* < 0.05] and again no interaction was detected. Independent *t*-tests revealed that methylone compared with vehicle did not affect ASR at any of the doses tested (Table 2).

**Figure 4:**
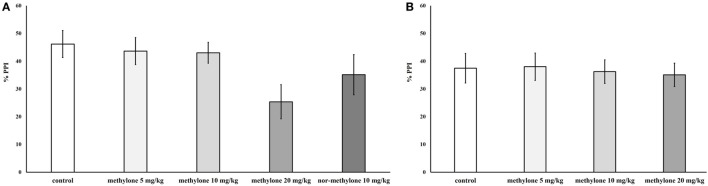
Mean percentage prepulse inhibition of methylone (5, 10, and 20 mg/kg) and nor-methylone (10 mg/kg) 15 min **(A)** and 60 min **(B)** after administration. Columns represent means and vertical bars SEMs.

Independent ANOVA showed a significant main effect of drug treatment on PPI [*F*_(3, 72)_ = 2.88, *p* < 0.05], no other interactions were observed. Subsequent independent *t*-test revealed a trend to decrease for 20 mg/kg at 15 min, compared with control, *t*(18) = 1.91, *p* = 0.1 (one-tailed). Nor-methylone did not differ from vehicle (Figure 4).

### The Effect of Methylone on Body Temperature

The effect of methylone on body temperature was analyzed using 3 × 2 × 13 mixed factorial ANOVAs with drug treatment (methylone at 10 and 20 mg/kg versus vehicle) and home-cage conditions (individually and group-housed rats) as independent factors, and time as a repeated measures factor. Mauchly’s test of sphericity was significant and Greenhouse–Geisser correction are presented for repeated measures, Mauchly’s *W*(44) = 0.05, *p* < 0.001. Although temperature data before drug administration were significantly different from vehicle, these data were averaged for individual treatment and subtracted from temperature data after drug administration.

Temperature data showed a significant main effect of drug treatment [*F*_(2, 54)_ = 5.29, *p* < 0.05], home-cage conditions [*F*_(1, 54)_ = 4.41, *p* < 0.05], and time [*F*_(5, 289)_ = 161.58, *p* < 0.001]. The interaction of drug treatment × time [*F*_(11, 289)_ = 6.87, *p* < 0.001] and the three-way interaction of drug treatment × time × home-cage conditions [*F*_(11, 289)_ = 4.3, *p* < 0.001] were significant.

**Figure 5:**
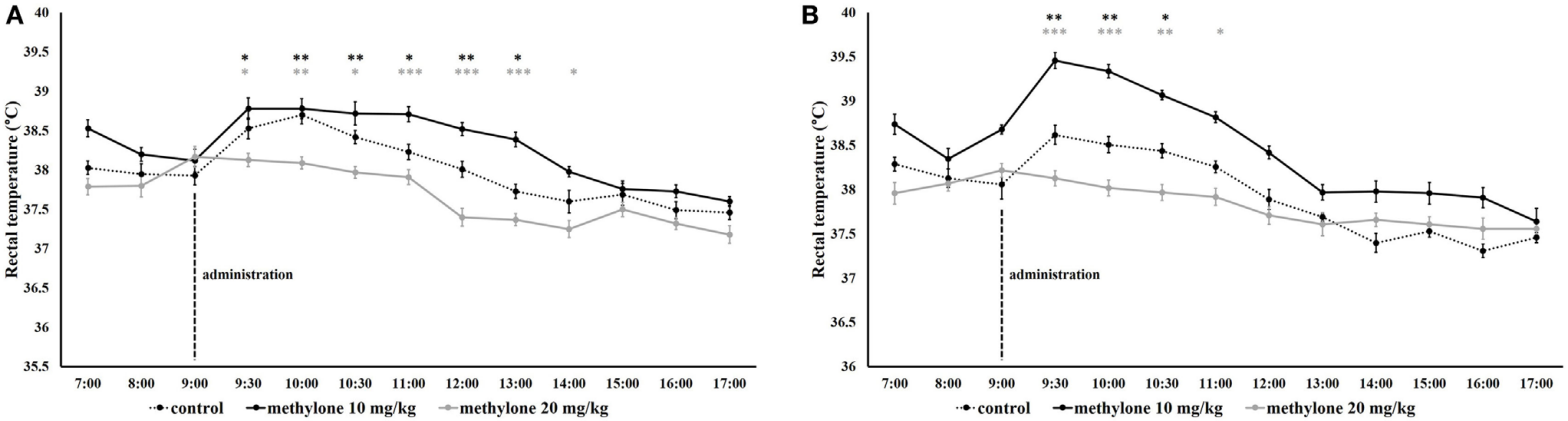
The effect of methylone on rectal temperature in individually **(A)** and group-housed **(B)** rats. Vertical lines represented administration of methylone (10 and 20 mg/kg or vehicle). Symbols represent means and vertical bars SEMs. ***p < 0.001, **p < 0.01, and *p < 0.05, gray asterisks refer to methylone (10 mg/kg) versus vehicle comparison, black asterisk methylone (20 mg/kg) versus vehicle comparison.

Independent *t*-tests revealed that under individually conditions, methylone significantly increased body temperature half an hour (9.30 h) after administration for both doses (10 and 20 mg/kg), an effect that was maintained until 13.00 for 10 mg/kg groups, minimum *t*(18) = 2.15, *p* < 0.05, and to 14.00 in the case of 20 mg/kg, minimum *t*(18) = 2.07, *p* = 0.05, Figure 5A.

In rats housed under group-housed condition, the temperature started to increase at 30 min after methylone administration after each of the doses. Methylone 10 mg/kg significantly increased body temperature from 9.30 to 10.30 h, minimum *t*(18) = 2.6, *p* < 0.05. At 20 mg/kg dose, temperature maintained elevated until 11.00, minimum *t*(18) = 2.46, *p* < 0.05, Figure 5B.

## Discussion

The main findings were as follows: methylone (i) had fast pharmacokinetics with a peak at 30 min, readily crossed the blood–brain barrier and reached levels approximately five times higher in the brain tissue (compared with serum); the major metabolite nor-methylone peaked in the brain at 30 min after methylone administration; (ii) showed marked stimulant effects at 15 min after administration which significantly diminished when tested 1 h after administration; (iii) methylone has relatively weak potency to disrupt PPI; and (iv) methylone significantly increased rectal temperature in individually as well as group-housed rats. When nor-methylone was administered alone, even though it reached approximately 1/2 and 1/3 of the serum and brain levels compared with methylone, it had comparable stimulant potency to methylone.

### Pharmacokinetics

Compared with our data, Elmore et al. (32) found the peak serum of methylone levels even earlier at 15 min using the same route of administration. Interestingly, Lopez-Arnau et al. (33) found maximum plasma levels at 30 min after oral administration of methylone in rats which is indicative of a very fast gastrointestinal absorption. Additionally, only our experiments indicate a very fast and effective crossing of blood–brain barrier as methylone levels in the brain were more than five times those in serum. The incorporation of methylone into the brain may be associated with high lipophilicity, as we have already suggested for other compounds, e.g., PMMA or MDAI (5,6-methylenedioxy-2-aminoindane) (21, 27). Similarly, as methylone, its metabolite nor-methylone showed similar serum:brain ratio. The other important and major metabolites 4-OH-MeO-MC (4-hydroxy-3-methoxymethcathinone) (1, 34) were also detected in serum and brain. The rapid decrease of its dominance in the analytical spectrum at 60 min may be related to its fast conjugation, which would explain its lower plasmatic concentrations compared with nor-methylone. Even though we did not perform enzymatic hydrolysis, we might assume that the rapid decrease of its levels might be related to its fast conjugation with glucuronic and/or sulfuric acid that is typical for fenolic metabolites (35). Compared with MDMA where peak MDMA concentrations are achieved within 1 h after subcutaneous or oral administration both methylone and its metabolite nor-methylone showed a more rapid kinetic profile (36, 37) which is in line with the reported shorter duration of effects in humans (and might lead to more frequent re-dosing by users).

### Acute Toxicity

According to our knowledge, there is no evidence about determination of lethal methylone dose in animals. In our study, we obtained unexpected findings on the lethal effects of the highest dose of methylone (40 mg/kg) in the rats. The symptoms observed in this case (i.e., hyperventilation, seizures) were similar to symptoms detected in MDAI (27) and may be associated with serotonin syndrome, mainly hyperthermia which is one of the core symptom caused by 5-HT release (38).

### Open Field

In accordance with its kinetic profile, the overall locomotor stimulatory activity was more pronounced 15 min after administration and was also comparable with other studies in rats (16, 19) and mice (17, 18). In mice, after methylone 30 mg/kg, locomotor activity was lower compared with 10 mg/kg (18) indicative of an inverted U shaped curve of locomotor effects. This inverted U shaped locomotor curve is also typical for most of the stimulants and characteristically linked to an increase in stereotyped behavior (e.g., circling) (21, 39). It is well established that the stimulatory versus hallucinogenic potency of cathinones and other related compounds is related to their DAT:SERT inhibition ratio. As stated above, methylone has been reported to have similar DAT:SERT inhibition ratio to cocaine (3, 12), and in contrast to other related cathinones, e.g., mephedrone, naphyrone, and methylenedioxypyrovalerone (MDPV) methylone has lower selectivity over DAT making it less stimulatory (3, 40). As reported in comparable behavioral studies of our currently submitted manuscripts, its stimulatory potency is slightly lower compared with mephedrone and much less potent compared with MDPV (unpublished observations Horsley et al.and Sichova et al.).

According to the temporal and spatial patterns in locomotor activity, methylone disrupted habituation, increased exploration, and stimulated activity at lower doses, however, high doses induced stereotyped behavior. In this respect, methylone behaves in a very similar manner to other stimulants and entactogens tested in identical (or near-identical) paradigms in our laboratory (21, 31).

### Prepulse Inhibition

Methylone has a relatively weak potency to disturb sensorimotor processing. In line with this, our recent experiments with mephedrone, nor-mephedrone, and MDPV showed comparable weak or negative effects on PPI in rats (unpublished observations Horsley et al. and Sichova et al.). Interestingly amphetamine, which is approximately 10 times more potent in disrupting PPI in rodents, in humans also failed to have disruptive effect on PPI (41). However, this might be related to the fact that it was used in much lower dose (0.45 mg/kg) in humans compared with rodents (typically 1–4 mg/kg). On the contrary drugs affecting mainly SERT, e.g., MDMA, PMMA, or MDAI seem to have much stronger ability to disrupt sensorimotor gating in animals as well as in humans (26, 31, 42). Since PPI is typically used as a model of psychotomimetic potential in animals with translational validity, we may conclude that methylone has only mild psychotomimetic effects. Apart from PPI, and similarly like with the open field, the habituation to startle was attenuated during the peak of methylone effect (i.e., in 15 min time of administration). This can be theoretically also related to the overall stimulatory effect or to anxiety, since with the highest dose also the decreased time spent in the center was present.

### Temperature

As expected and in accordance with previous studies with methylone (22, 43, 44), MDMA (45, 46) as well as our comparable studies with phenethylamine PMMA, and aminoindane MDAI (21, 27) the hyperthermic reaction was more pronounced in group-housed condition where it increased up to 1.5°C. The temperature increase was rapid and was not associated with visible perspiration as has been described with PMMA and MDAI (21, 27). In animals housed separately, the increase in temperature lasted for a 1 h longer compared with animals housed in groups. This is surprising since the opposite would be expected. This might be explained by accelerated metabolism due to higher increase in body temperature (cca 1°C) in animals housed in groups. Serotonergic drugs have more pronounced hyperthermic effects compared with drugs with dopaminergic actions. Since serotonin is a critical neuromodulator involved in the thermoregulation, with 5-HT2A receptors being a key mechanism responsible for hyperthermia (47). It is therefore very probable that this is also the case for methylone where the 5-HT2A receptor is stimulated *via* indirect mechanisms related to the increased serotonergic tone (44). On the other hand, in study of Javadi-Paydar et al. (19) was shown that mean body temperature did not vary more than 0.5°C from baseline temperature after methylone application. These findings of different (negative) results could be caused by methodological differences, where they measured temperature using radiotelemetry with lower doses of methylone than us.

Also in some of the cathinones, e.g., mephedrone have been also reported to induce hypothermia in rats but not mice (43, 48, 49). This effect is typically stimulated by activity at 5-HT1A receptors, and sometimes drugs that induce serotonin release might have biphasic effects on temperature or bidirectional depending on pharmacodynamics and the stimulation of these receptors (50).

Since here rats in group-housed conditions exhibited greater elevations in temperature (than under individually housed rats), this provides more support for the idea that environmental conditions that are crowded and/or hot (e.g., people dancing in a crowded clubs) increase the risk of hyperthermia and acute toxicity associated with methylone (51).

## Conclusion

Methylone and its primary metabolite, nor-methylone induced behavioral, and temperature changes that are comparable with MDMA and other related stimulants, however, our results indicate it has a weaker capacity to disrupt PPI than MDMA and other stimulants. Since we have observed lethal toxicity in our study and that several deaths have been also associated with methylone in humans, its toxicity should not be underestimated, especially when hyperthermic reaction appears in a crowded environments.

## Ethics Statement

All procedures were conducted in accordance with the principles of laboratory animal care of the National Committee for the Care and Use of Laboratory Animals (Czech Republic), and according to Guidelines of the European Union (86/609/EU). The protocol was approved by the National Committee for the Care and Use of Laboratory Animals (Czech Republic) under the number: MEYSCR-27527/2012-31.

## Author Contributions

All authors made a significant contribution to this study, read, revised, and gave final approval for the current version of the work to be published.

## Conflict of Interest Statement

The authors declare that the research was conducted in the absence of any commercial or financial relationships that could be construed as a potential conflict of interest.
